# Antibiotics Disturb Dentin Formation and Differentiation of Dental Pulp Stem Cells: The Role of Microbiota in Cellular Turnover of Mouse Incisor

**DOI:** 10.1155/2020/5154707

**Published:** 2020-09-17

**Authors:** Shenping Su, Yi Ren, Yi Zhang, Yuming Zhao, E. Xiao

**Affiliations:** ^1^Department of Pediatric Dentistry, Peking University School and Hospital of Stomatology, China; ^2^Department of Oral and Maxillofacial Surgery, Peking University School and Hospital of Stomatology, China

## Abstract

Dentin formation was dependent on osteo-/odontogenic differentiation of dental pulp stem cells (DPSCs). It was observed in previous studies that antibiotic treatment in a clinical and animal model resulted in impaired mineralization of dental tissues. We previously reported that microbiota maintained the function of bone marrow mesenchymal stem cells, while whether microbiota dysbiosis caused by antibiotic treatment contributed to DPSCs dysfunction and impaired dentin formation is still not known. In this study, we aimed to clarify the role of microbiota or its metabolic products on dental mineralization and the function of DPSCs. Mice were treated with antibiotics to disrupt microbiota; then, the growth rate and histological characteristics of incisors as well as the biological characteristics of DPSCs *in vitro* were compared with specific pathogen-free (SPF) mice. In antibiotic-treated mice (AbT), we found a diminished quantity of microbiota and reduced growth rate of mechanical injured incisor, as well as decreased colony-forming rate and impaired ability of osteo-/odontogenic differentiation of DPSCs, in comparison to SPF mice. Colonization of AbT mice with SPF mice replanted the microbiota by cohousing (conventionalized (ConvD)) and normalized the growth rate of injured incisors and colony-forming and osteo-/odontogenic differentiation ability of DPSCs. Giving short-chain fatty acids (SCFAs) by oral gavage after antibiotic treatment also rescued the growth rate of incisors and the differentiation ability of DPSCs and enhanced proliferation ability of DPSCs. Collectively, gut microbiota could make contribution to maintain continuous growth of injured rodent incisor and differentiation capacity of DPSCs; SCFAs might play a crucial role in this process.

## 1. Introduction

Antibiotics are by far the most common medications prescribed for children. Studies have suggested the association between early antibiotic use and disease phenotypes in adulthood. Many clinical studies have found that antibiotic use during pregnancy or early childhood growth is associated with an increased risk of molar-incisor hypomineralization (MIH) [1–4], suggesting that antibiotics may lead to abnormal mineralization of dental hard tissues. However, the mechanism of antibiotics affecting the mineralization of dental hard tissues has not been reported.

Studies have confirmed that the use of antibiotics can disrupt gut microbiota and contribute to the occurrence and development of various diseases [5]. Gut microbiota functioned as a hidden organ, altering host metabolism and shaping host systemic immune function [6, 7], and consequently played an important role in brain, lung, liver, and cardiovascular system homeostasis and so on distantly [8–11]. Furthermore, the dysbiosis of microbiota in diseases such as diabetes or antibiotic treatment also caused organ or tissue dysfunction [12, 13], via the alteration of pathogen-associated molecular patterns (PAMPs) including agents like peptidoglycans and lipopolysaccharide (LPS) or metabolites of microbiota such as short-chain fatty acids (SCFAs).

Former studies have shown the impact of microbiota on hard tissue such as bone. The absence of gut microbiota in germ-free (GF) mice led to increased bone mass [14], which could be duplicated in antibiotic-treated (AbT) mice [13, 15]. GF mouse-derived bone marrow mesenchymal stem cells (BMMSCs) showed higher colony-forming ability, proliferation rate, and increased osteogenic ability, and microbiota replantation could normalize the proliferation and differentiation abilities of BMMSCs [16]. To some extent, the characteristics of rodent incisors are similar with bone, featured with continuous growing and remodeling, and mesenchymal stem cells play a critical role in growing and remodeling process. BMMSCs contribute to the regeneration and reparation of bone, and dental pulp stem cells (DPSCs) play an important role in the regeneration of dentin-pulp-like complex [17]. *In vitro*, DPSCs shared similar characteristics with BMMSCs, including proliferation and multidifferentiation abilities [18, 19].

Therefore, in this study, we hypothesized that combined antibiotic treatment caused dysbiosis of gut microbiota, resulting in the alteration of the production of metabolites such as SCFAs or PAMPs such as lipopolysaccharide (LPS), and would impair the cellular turnover and continuous growth of mouse incisors. Thus, in the present study, we compared the growth rate, the histological characteristics of dentin, and the characteristics of dental pulp stem cells of mouse incisors in different conditions of gut microbiota.

## 2. Materials and Methods

### 2.1. Animal Model

The protocol of animal experiments had been reviewed and approved by the Ethics Committee of the Peking University Health Science Center (No. LA2018184). Sixty 3-week-old C57BL/6 female mice were brought into research and randomly divided into 5 groups: specific pathogen-free group (SPF), antibiotic treatment group (AbT), conventionalized group (ConvD), lipopolysaccharide group (LPS), and short-chain fatty acid group (SCFA). The mice in the AbT group were given antibiotic water containing ampicillin 1 g/L, neomycin 1 g/L, metronidazole 1 g/L, and vancomycin 0.5 g/L, while the mice in the SPF group were fed with sterile distilled water as control. After 3 weeks, the mice in the AbT group cohabited with mice in the SPF group for 1 week and defined as the ConvD group. Besides, mice received short-chain fatty acid solution (sodium acetate, sodium propionate, sodium butyrate, 150 mM, Sigma-Aldrich, St. Louis, MO, USA) and lipopolysaccharide (50 *μ*g/ml, Sigma-Aldrich, St. Louis, MO, USA) through oral gavage named the SCFA and LPS group, respectively.

### 2.2. Mechanical Injury

Mice were anesthetized by intraperitoneal injection of 4% chloral hydrate (Hushi, Shanghai, China). Clipping of the incisor of each mouse was performed on the right lower incisor, by removing about 2 mm from the tip of the erupted part using a high-speed diamond cylinder bur [20]. The pictures of incisors were taken by the same device every 24 hours for 3 days, and the height differences were analyzed by ImageJ.

### 2.3. Histological Analysis

To investigate the histological characteristics in different groups after clipping the incisor, mice were sacrificed by neck breaking at 0, 12, 24, and 72 h after surgery. Hemimandibles were dissected, fixed with 10% formalin for 24 h, decalcified in 20% ethylene diamine tetraacetic acid (EDTA), and processed for paraffin embedding. 4-micrometer sections were obtained using the Leica cryostat (Germany) and processed for hematoxylin-eosin (H&E) staining and examined by a BX51 light microscope (Olympus, Tokyo, Japan).

DSPP and Ki-67 expression was evaluated by immunohistochemistry. In brief, sections were treated with antigen retrieval and incubated with mouse monoclonal anti-DSPP (Santa Cruz, sc73632, 1 : 50) and rabbit polyclonal anti-Ki67 (Abcam, ab15580, 1 : 100). Mouse-IgG binding protein-HRP and rabbit-IgG binding protein-HRP (Zsbio) were added, and a DAB kit was used to detect primary antibodies.

### 2.4. Primary Cultures

The pulp of intact incisors was obtained from the mouse upper incisor, and dental pulp stem cells (DPSCs) were isolated. The tissue was minced into 0.5 mm pieces, transferred into a T25 culture flask (Corning, USA), and incubated with *α*-modified minimum essential medium (*α*-MEM, GIBCO/BRL) with 20% fetal bovine serum (FBS, GIBCO) containing ascorbic acid (10 mM, GIBCO) and glutamate (2 mM, GIBCO) at 37°C with 5% CO_2_. Osteo-/odontogenic differentiation medium consisted of basal medium plus 10 nM dexamethasone, 50 *μ*g/ml ascorbic acid 2-phosphate, and 10 mM *β*-glycerophosphate (Sigma-Aldrich, St. Louis, MO, USA).

### 2.5. Real-Time Polymerase Chain Reaction

Total RNA was extracted from DPSCs using TRIzol reagent (Invitrogen Life Technologies, CA, USA). Complementary DNAs (cDNAs) were prepared using the GoScript Reverse Transcription System (Promega, Madison, WI, USA). Real-time polymerase chain reaction (real-time PCR) was performed with an ABI Prism 7500 (Applied Bioscience, Foster City, CA, USA). *β*-Actin was used to normalize gene expression, and the relative mRNA expression levels were calculated. Primers used in this study are shown in Table 1.

### 2.6. Western Blot

After 7-day osteogenic-induced medium treatment, whole cell lysates of DPSCs were extracted by using RIPA lysis buffer (Solarbio). 40 mg of protein from each sample was resolved by 10% sodium dodecylsulfate–polyacrylamide gel electrophoresis under reducing conditions, separated on 10% SDS-PAGE gels, and transferred to a polyvinylidene difluoride membrane (PVDF, Immun-Blot, Bio-Rad Laboratories). The membranes were blocked with 5% skim milk and immunoblotted with affinity-purified goat polyclonal anti-mouse DSPP and GAPDH (Santa Cruz Biotechnology, Santa Cruz, CA), and bands were visualized using Fusion Fx (Vilber Lourmat, France).

### 2.7. Statistical Analysis of Data

Statistical analysis was performed by GraphPad Prism 6 software using one-way ANOVA, and statistical significance was determined at *P* ≤ 0.05.

## 3. Results

### 3.1. Mouse Injured Incisors Showed Lower Growth Rate after Antibiotic Treatment

**Figure 1:**
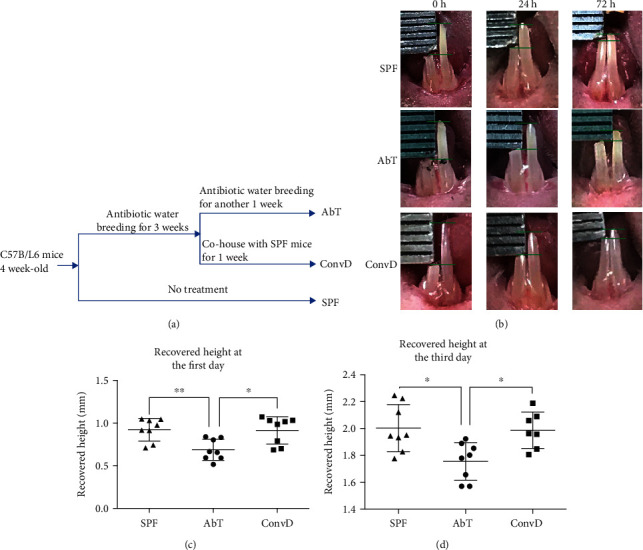
Antibiotic treatment decreased the growth rate of mouse incisors. (a) Experimental procedure schematic. (b) Representative images of height difference of bilateral incisors at different time points after mechanical injury. (c) Histogram of statistical analysis of recovered height of incisors at a 24-hour time point. (d) Histogram of statistical analysis of recovered height of incisors at a 72-hour time point. Error bars represent the SEM from the mean values. ^∗∗^*P* < 0.001; ^∗^*P* < 0.05. AbT: antibiotic treated; ConvD: conventionalized; SPF: specific pathogen-free.

Firstly, we compared the growth rate of incisors. 2 mm of the right incisor was clipped in SPF, AbT, and ConvD mice (Figure 1(a)); then, the height difference of bilateral lower incisors was measured every 24 h (Figure 1(b)). The results showed that mechanically injured incisors grew rapidly in the first 24 h and almost 50 percent of the shortened height recovered. The growth rate of SPF mice was the highest, which was 0.9218 ± 0.04682 mm/24 h (*n* = 8), followed by the ConvD group (0.9139 ± 0.05681 mm/24 h, *n* = 8), and the growth rate of AbT mice was significantly reduced (0.6869 ± 0.04357 mm/24 h, *n* = 8) (Figure 1(c)). At 72 h after injury, the disparity between the AbT (1.756 ± 0.04949 mm/72 h) and SPF (2.003 ± 0.06200 mm/72 h) group was reduced and the SPF and ConvD (1.987 ± 0.05116 mm/72 h) group still exhibited the highest growth rate (Figure 1(d)).

### 3.2. Delayed Dentin Mineralization in Antibiotic-Treated Mice

**Figure 2:**
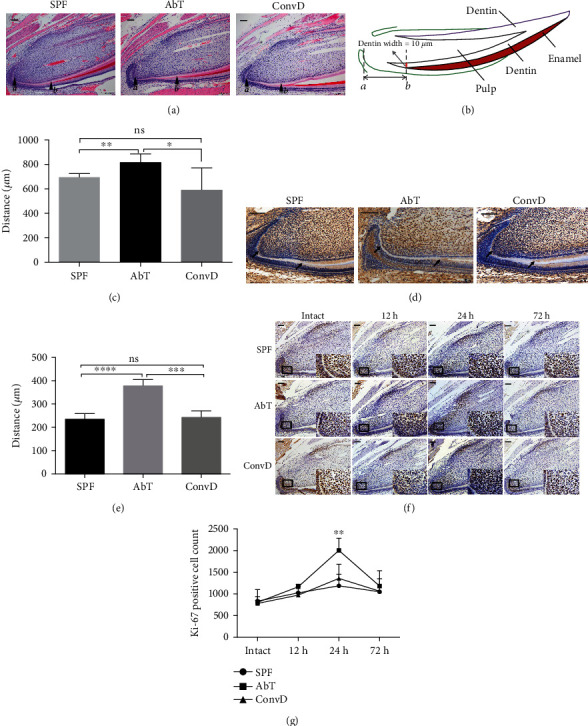
Histological analysis of the apical area and dentin formation of incisors. (a) H&E staining showed the histological structure of the apical area. (b) The distance was measured as the sketch map has shown; point *a* stands for epithelial invagination, and point *b* stands for mineralized dentin reaching a width of 10 *μ*m. (c) The distance between points *a* and *b* was calculated, and it showed a longer distance in AbT. (d, e) Immunohistochemical staining of DSPP showed delayed mineralization of dentin in the AbT group. (f, g) Immunohistochemical staining of Ki-67 showed that at 12 hours after injury, Ki-67-positive cells began to accelerate (the second panel), and acceleration of Ki-67-positive cells reached the peak at 24 hours after injury (the third panel), and at a 72-hour time point, the amount of Ki-67-positive cells descended to almost the same level as the 12-hour time point (the right panel) (bar = 100 *μ*m). All experimental data were verified in at least three independent experiments. Error bars represent the SEM from the mean values. ^∗∗∗∗^*P* < 0.0001; ^∗∗∗^*P* < 0.001; ^∗∗^*P* < 0.01; ^∗^*P* < 0.05. AbT: antibiotic treated; ConvD: conventionalized; SPF: specific pathogen-free.

In order to further clarify the reason for the slower growth of the incisor in the AbT group, dentin formation, cell proliferation, and odontoblast differentiation were analyzed. The results of HE staining showed a longer distance from the epithelial invagination (point *a*) to mineralized dentin (point *b*) in the AbT group (815.5 ± 31.49 *μ*m), compared to the SPF group (695.7 ± 13.31 *μ*m) and the ConvD group (588.5 ± 81.79 *μ*m) (Figures 2(a)–2(c)). This result indicated that the mineralization was delayed in the AbT group.

Immunohistochemistry analysis showed that the dental pulp, especially the odontoblast layer, expressed DSPP, which is a marker of odontoblast cells. Moreover, the beginning location of DSPP expression was further away from the apical foreman in the AbT group (377.7 ± 13.60 *μ*m), compared to the SPF group (234.4 ± 12.32 *μ*m) and the ConvD group (242.7 ± 13.69 *μ*m) (Figures 2(d) and 2(e)).

It was generally considered that the growth rate of mouse incisors was related to cell proliferation at the apical end, so cell proliferation was examined histologically. Immunohistochemical staining showed that Ki-67-positive (Ki-67(+)) cells aggregated in the apical region in all three groups at 12 h after mechanical injury and reached the peak level at 24 h. Moreover, at 24 h time point, there were more Ki-67-positive cells expressed in the AbT group (2005 ± 199.5 cells), compared to the SPF group (1188 ± 191.0 cells) and the ConvD group (1360 ± 229.5 cells), and the latter two groups showed no significant difference (Figures 2(f) and 2(g)). The above results suggested that the slow dentin formation is not due to proliferation but differentiation of DPSCs.

### 3.3. DPSCs Exhibited Impaired Mineralized Potential after Antibiotic Treatment

**Figure 3:**
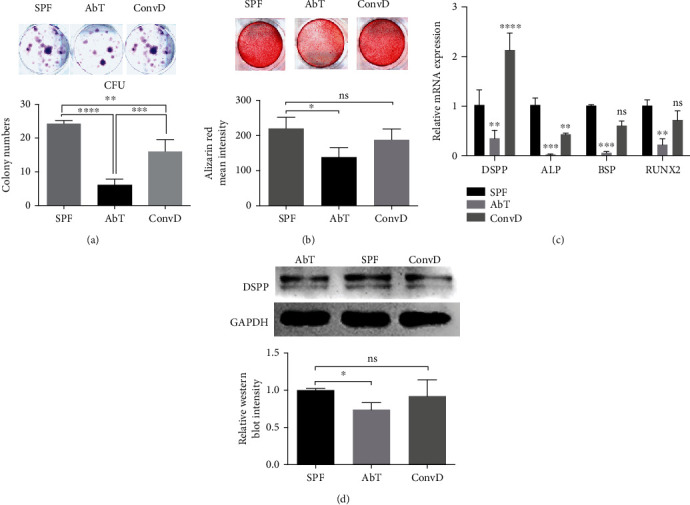
DPSCs from AbT mice exhibited impaired colony-forming ability and mineralized potential. (a) Colony-forming unit experiments showed that DPSCs derived from AbT mice had lower colony-forming rate compared to those from SPF and ConvD mice. (b) Alizarin red staining showed that AbT-derived DPSCs formed less mineralized nodule *in vitro* compared to SPF and ConvD groups. (c) Real-time PCR results showed that SPF-DPSCs exhibited higher expression of mineralization relative mRNA. (d) Western blot analysis showed that AbT-DPSCs expressed lower DSPP after osteo-/odontogenic induction *in vitro*. All experimental data were verified in at least three independent experiments. Error bars represent the SEM from the mean values. ^∗∗∗∗^*P* < 0.0001; ^∗∗∗^*P* < 0.001; ^∗∗^*P* < 0.01; ^∗^*P* < 0.05. AbT: antibiotic treated; ConvD: conventionalized; SPF: specific pathogen-free; DSPP: dentin sialophosphoprotein.

The changes of mineralization of DPSCs were further confirmed *in vitro*. We isolated and compared the characteristics of DPSCs, especially proliferation and odontoblast differentiation. The result of the colony-forming assay showed that DPSCs derived from AbT mice exhibited lower colony-forming rate, with 6.20 ± 0.7348 colonies/5000 cells, compared to DPSCs derived from SPF mice (24.25 ± 0.4787 colonies/5000 cells) and ConvD mice (16.00 ± 1.581 colonies/5000 cells) (Figure 3(a)). Alizarin red staining showed that AbT-derived DPSCs formed less mineralized nodule *in vitro* compared to the SPF group (138.7 ± 15.36 vs. 219.9 ± 18.52, respectively) (Figure 3(b)). The results of real-time PCR showed that the mRNA expression levels of *DSPP*, *ALP*, *BSP*, and *RUNX2* mRNA were lower in AbT-derived DPSCs (Figure 3(c)). The results of western blot showed similar tendency that the expression level of DSPP was lower in AbT-derived DPSCs when compared with SPF-derived and ConvD-derived DPSCs (Figure 3(d)).

### 3.4. SCFAs but Not LPS Rescued Antibiotic-Induced Compromised Incisor Growth Rate and Proliferation and Mineralization Capacity of DPSCs

**Figure 4:**
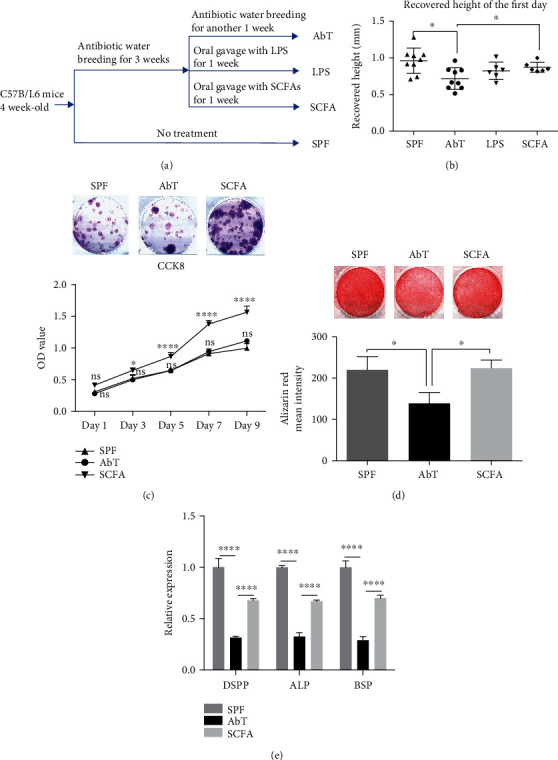
SCFAs but not LPS could rescue the mouse incisor growth rate and reinforced characteristics of DPSCs after antibiotic treatment. (a) Experimental procedure schematic. (b) The result of recovered height of injured incisors at the first day showed significant reduction in AbT compared to SPF and SCFA gavage, while LPS gavage showed no difference when compared with AbT. (c) DPSCs derived from SCFA mice showed greater colony-forming and proliferative capacity *in vitro*. (d) Alizarin red staining showed that SCFA-DPSCs formed more mineralized nodule than AbT DPSCs, and there was no significant difference between SCFA and SPF. (e) Real-time PCR results showed that the expression of *DSPP*, *ALP*, and *BSP* was higher in SCFA compared to that of AbT, while SPF showed the highest expression of mRNA above. All experimental data were verified in at least three independent experiments. Error bars represent the SEM from the mean values. ^∗∗∗∗^*P* < 0.00001; ^∗∗^*P* < 0.01; ^∗^*P* < 0.05. AbT: antibiotic treated; ConvD: conventionalized; SPF: specific pathogen-free; DSPP: dentin sialophosphoprotein; ALP: alkaline phosphatase; BSP: bone sialoprotein.

The following question is how microbiota affect dentin, so we replenished LPS or SCFA instead of cohousing with SPF mice (Figure 4(a)). The results showed that after LPS oral gavage, there was no difference in growth rate compared to the AbT group (0.8235 ± 0.04873 mm/24 h vs. 0.7176 ± 0.04917 mm/24 h), while the growth rate of injured mouse incisors in the SCFA group (0.8733 ± 0.02799 mm/24 h) was significantly higher compared to that in the AbT group (Figure 4(b)). Furthermore, DPSCs derived from SCFA mice showed greater colony-forming and proliferative capacity *in vitro* (Figure 4(c)). DPSCs derived from the SCFA group also formed more mineralized nodule (233.7 ± 14.4) compared to the AbT group (138.7 ± 15.36) (Figure 4(d)), and osteo-/odontogenic-related mRNA were upregulated (Figure 4(e)). These results proposed that the metabolic products of microbiota may play a pivotal role in distal target tissue.

## 4. Discussion

In the present study, we showed that the osteo-/odontogenic differentiation potential of DPSCs, dentin mineralization, and continuous growing of mouse incisors were impaired in antibiotic-treated mice, which were characterized by reduced quantity of gut microbes. The microbiota replantation and the supplement of SCFAs could rescue impaired dentin formation. This study verified the hypothesis for the first time that antibiotic-induced dysbiosis of gut microbiota impaired the turnover of DPSCs and continuous growth of mouse incisors via SCFA.

To determine the importance of gut commensal microbiota in dentin formation and characteristics of DPSCs, mice were subjected to a 4-week oral administration of antibiotic combination [21]. It was reported that after 4 weeks of antibiotic treatments, the abundance of intestinal microbiota decreased significantly [22]. In this study, it was verified that more than 99% bacteria were depleted after antibiotic treatment by real-time PCR, using universal 16S rRNA primers (Figure S1). And after cohousing for 1 week, the quantity of gut microbiota recovered in the ConvD group (Figure S1).

In a mouse incisor, it has been suggested that the odontoblasts which arise from DPSCs participated in the continuous dentin formation [23]. During the process of continuous growth, stem cells in the proximal end of the incisor gave rise to a spatially distinct transit-amplifying cell (TAC) population of rapidly proliferating cells that differentiated into the main specialized tooth-specific cell type, odontoblasts, and the fibroblastic pulp cells [24] which continuously produced dentin in the apical. The newly formed dentin moved apically together with the odontoblasts to compensate the wearing at the cut end [25]. In this study, we found that the delayed incisor growth in AbT mice was accompanied by delayed DSPP expression *in vivo* and impaired DPSCs osteo-/odontogenic differentiation *in vitro*. At the same time, Ki-67 staining increased. This proposed that the differentiation of TAC and the turnover of odontoblasts were interrupted by gut dysbiosis which resulted in delayed dentin formation and impaired continuous growth of incisors. Then, impaired osteo-/odontogenic differentiation in turn caused increased Ki-67-positive TAC cells. Taken together, we assumed that the decreased growth rate of incisors in AbT mice was related to delayed dentin mineralization.

The development of gut dysbiosis can set into processes that activate the host immune and inflammation response and cause metabolic abnormalities. On one hand, the microbiota plays a fundamental role on the induction, training, and function of the host immune system. Their production of PAMPs such as LPS fuels the inflammatory process in the intestinal mucosa, and systemic entry of PAMPs can affect many distant organs [26]. On the other hand, metabolites of microbiota such as SCFAs, including acetate, butyrate, and propionate, are major fermentation products of microorganisms in the gut. SCFAs are the main energy source for colonocytes that also provide several other beneficial effects in maintaining intestinal homeostasis. Besides, they can affect distal organs [27]. To find out how did microbiota affect dentin formation, representative metabolites (SCFAs) and PAMPs (LPS) were chosen to supply to mice that received antibiotics for 3 weeks, respectively. The present results revealed that SCFA supplement by oral gavage but not LPS could rescue the growth rate of the incisor *in vivo* and the proliferative and osteo-/odontogenic ability of DPSCs of AbT mice *in vitro*.

Short-chain fatty acids (SCFAs) are microbial metabolites, produced by fermentation of nondigestible substrates, and the major components contain acetate, propionate, and butyrate [28]. Compared to the case in conventionally raised mice, the contents of the colons of germ-free mice included more of the nondigestible plant oligosaccharide raffinose, a bacterial fermentation substrate, and markedly diminished amounts of SCFAs. Antibiotics also altered the short-chain fatty acid production via the microbiota. It is reported that fecal concentrations of SCFAs are reduced in patients receiving antibiotics [29]. SCFAs were transported from the intestinal lumen into the blood across the colonocytes [30] and participated in the progress of several diseases such as obesity and diabetes [31]. In this study, the SCFA levels in serum were measured via GC-MS. We found that after antibiotic treatment, the amount of acetate acids and butyrate acid was reduced (Figure S2), which was consistent with a previous study [15, 32]. A previous study declared that SCFA supplementation suppressed bone mass in antibiotic-treated mice, suggesting that SCFAs could rescue the influence of antibiotics on the bone marrow [15]. Another study also showed that butyrate facilitated the osteogenic differentiation of dental follicle cells in early stages [33]. Our study showed that SCFA supplementation increased incisor growth rate which is finally comparable to SPF mice, probably resulting from the enhanced proliferative and odontogenic differentiation abilities of DPSCs derived from the SCFA group. However, it is not clear how SCFAs contribute to tooth growth, and the mechanisms need to be further elucidated.

## 5. Conclusion

In this study, antibiotic complex-depleted microbiota resulted in delayed dentin formation, odontoblast differentiation, and impaired colony-forming as well as osteo-/odontogenic differentiation capacity of DPSCs. Microbiota replantation and SCFAs supplement could rescue the growth rate of incisors and characteristics of DPSCs after antibiotic complex treatment.

## Figures and Tables

**Table 1:** Primers used for real-time PCR.

Target gene	Sequence
*β-Actin*	Forward: GTGACGTTGACATCCGTAAAGA
	Reverse: GCCGGACTCATCGTACTCC
*DSPP*	Forward: AACTCTGTGGCTGTGCCTCT
	Reverse: TATTGACTCGGAGCCATTCC
*ALP*	Forward: CTATCCTGGCTCCGTGCTC
	Reverse: GCTGGCAGTGGTCAGATGTT
*BSP*	Forward: AAAGTGAAGGAAAGCGACGA
	Reverse: GTTCCTTCTGCACCTGCTTC
*RUNX2*	Forward: GACTGTGGTTACCGTCATGGC
	Reverse: ACTTGGTTTTTCATAACAGCGGA

## Data Availability

The in vivo and in vitro data used to support the findings of this study are included within the article or the supplementary materials.

## Supplementary Materials

Supplementary Materials Figure S1: validation of bacterial depletion efficiency after antibiotic treatment. (A) Standard curve of the CT value vs. the amount of bacteria. (B) Genomic DNA was isolated from equal amounts of fecal material of SPF, AbT, and ConvD mice. 16S rRNA gene expression of total bacteria was determined by qPCR, using universal 16S rRNA primers. ^∗∗∗∗^*P* < 0.0001. Figure S2: serum concentration of SCFAs showed decreased levels of acetic, propionic, and butyric acid in AbT mice. ^∗∗^*P* < 0.01.

## References

[1] Laisi S., Ess A., Sahlberg C., Arvio P., Lukinmaa P. L., Alaluusua S. (2009). Amoxicillin may cause molar incisor hypomineralization. *Journal of Dental Research*.

[2] Wuollet E., Laisi S., Salmela E., Ess A., Alaluusua S. (2016). Molar-incisor hypomineralization and the association with childhood illnesses and antibiotics in a group of Finnish children. *Acta Odontologica Scandinavica*.

[3] Giuca M. R., Cappè M., Carli E., Lardani L., Pasini M. (2018). Investigation of clinical characteristics and etiological factors in children with molar incisor hypomineralization. *International Journal of Dentistry*.

[4] Kumazawa K., Sawada T., Yanagisawa T., Shintani S. (2012). Effect of single-dose amoxicillin on rat incisor odontogenesis: a morphological study. *Clinical Oral Investigations*.

[5] Sun J., Chang E. B. (2014). Exploring gut microbes in human health and disease: pushing the envelope. *Genes & Diseases*.

[6] Levy M., Kolodziejczyk A. A., Thaiss C. A., Elinav E. (2017). Dysbiosis and the immune system. *Nature Reviews. Immunology*.

[7] Rooks M. G., Garrett W. S. (2016). Gut microbiota, metabolites and host immunity. *Nature Reviews. Immunology*.

[8] Fung T. C., Olson C. A., Hsiao E. Y. (2017). Interactions between the microbiota, immune and nervous systems in health and disease. *Nature Neuroscience*.

[9] Budden K. F., Gellatly S. L., Wood D. L. A. (2017). Emerging pathogenic links between microbiota and the gut-lung axis. *Nature Reviews. Microbiology*.

[10] Minemura M., Shimizu Y. (2015). Gut microbiota and liver diseases. *World Journal of Gastroenterology*.

[11] Tang W. H. W., Kitai T., Hazen S. L. (2017). Gut microbiota in cardiovascular health and disease. *Circulation Research*.

[12] Xiao E., Mattos M., Vieira G. H. A. (2017). Diabetes enhances IL-17 expression and alters the oral microbiome to increase its pathogenicity. *Cell Host & Microbe*.

[13] Cho I., Yamanishi S., Cox L. (2012). Antibiotics in early life alter the murine colonic microbiome and adiposity. *Nature*.

[14] Sjogren K., Engdahl C., Henning P. (2012). The gut microbiota regulates bone mass in mice. *Journal of Bone and Mineral Research*.

[15] Yan J., Herzog J. W., Tsang K. (2016). Gut microbiota induce IGF-1 and promote bone formation and growth. *Proceedings of the National Academy of Sciences of the United States of America*.

[16] Xiao E., He L., Wu Q. (2017). Microbiota regulates bone marrow mesenchymal stem cell lineage differentiation and immunomodulation. *Stem Cell Research & Therapy*.

[17] Gronthos S., Mankani M., Brahim J., Robey P. G., Shi S. (2000). Postnatal human dental pulp stem cells (DPSCs) in vitro and in vivo. *Proceedings of the National Academy of Sciences of the United States of America*.

[18] Alge D. L., Zhou D., Adams L. L. (2010). Donor-matched comparison of dental pulp stem cells and bone marrow-derived mesenchymal stem cells in a rat model. *Journal of Tissue Engineering and Regenerative Medicine*.

[19] Yang M., Zhang H., Gangolli R. (2014). Advances of mesenchymal stem cells derived from bone marrow and dental tissue in craniofacial tissue engineering. *Current Stem Cell Research & Therapy*.

[20] Gomes J. R., Omar N. F., dos Santos Neves J., Narvaes E. A. O., Novaes P. D. (2010). Increase of MT1-MMP, TIMP-2 and Ki-67 proteins in the odontogenic region of the rat incisor post-shortening procedure. *Journal of Molecular Histology*.

[21] Rakoff-Nahoum S., Paglino J., Eslami-Varzaneh F., Edberg S., Medzhitov R. (2004). Recognition of commensal microflora by toll-like receptors is required for intestinal homeostasis. *Cell*.

[22] Ge X., Ding C., Zhao W. (2017). Antibiotics-induced depletion of mice microbiota induces changes in host serotonin biosynthesis and intestinal motility. *Journal of Translational Medicine*.

[23] An Z., Sabalic M., Bloomquist R. F., Fowler T. E., Streelman T., Sharpe P. T. (2018). A quiescent cell population replenishes mesenchymal stem cells to drive accelerated growth in mouse incisors. *Nature Communications*.

[24] Smith C. E., Warshawsky H. (1975). Cellular renewal in the enamel organ and the odontoblast layer of the rat incisor as followed by radioautography using 3H-thymidine. *The Anatomical Record*.

[25] Klevezal G. A., Pucek M., Sukhovskaja L. I. (1990). Incisor growth in voles. *Acta Theriologica*.

[26] Xiao S., Fei N., Pang X. (2014). A gut microbiota-targeted dietary intervention for amelioration of chronic inflammation underlying metabolic syndrome. *FEMS Microbiology Ecology*.

[27] Cox M. A., Jackson J., Stanton M. (2009). Short-chain fatty acids act as antiinflammatory mediatorsby regulating prostaglandin E2 and cytokines. *World Journal of Gastroenterology*.

[28] Valdes A. M., Walter J., Segal E., Spector T. D. (2018). Role of the gut microbiota in nutrition and health. *BMJ*.

[29] Beaugerie L., Petit J.-C. (2004). Antibiotic-associated diarrhoea. *Best Practice & Research Clinical Gastroenterology*.

[30] den Besten G., van Eunen K., Groen A. K., Venema K., Reijngoud D. J., Bakker B. M. (2013). The role of short-chain fatty acids in the interplay between diet, gut microbiota, and host energy metabolism. *Journal of Lipid Research*.

[31] Zhao L., Zhang F., Ding X. (2018). Gut bacteria selectively promoted by dietary fibers alleviate type 2 diabetes. *Science*.

[32] Brestoff J. R., Artis D. (2013). Commensal bacteria at the interface of host metabolism and the immune system. *Nature Immunology*.

[33] Drees J., Felthaus O., Gosau M., Morsczeck C. (2014). Butyrate stimulates the early process of the osteogenic differentiation but inhibits the biomineralization in dental follicle cells (DFCs). *Odontology*.

