# Supplementary material for: Antibiotics Disturb Dentin Formation and Differentiation of Dental Pulp Stem Cells: The Role of Microbiota in Cellular Turnover of Mouse Incisor
Source: Stem Cells Int. 2020 Sep 17;2020:5154707. doi: 10.1155/2020/5154707 (PMC7519450; doi:10.1155/2020/5154707)
Supplement: Supplementary Materials — Figure S1: validation of bacterial depletion efficiency after antibiotic treatment. (A) Standard curve of the CT value vs. the amount of bacteria. (B) Genomic DNA was isolated from equal amounts of fecal material of SPF, AbT, and ConvD mice. 16S rRNA gene expression of total bacteria was determined by qPCR, using universal 16S rRNA primers. ∗∗∗∗P < 0.0001. Figure S2: serum concentration of SCFAs showed decreased levels of acetic, propionic, and butyric acid in AbT mice. ∗∗P < 0.01. [file 5154707.f1.pdf]

**Supplementary Material**

**Figure S1**

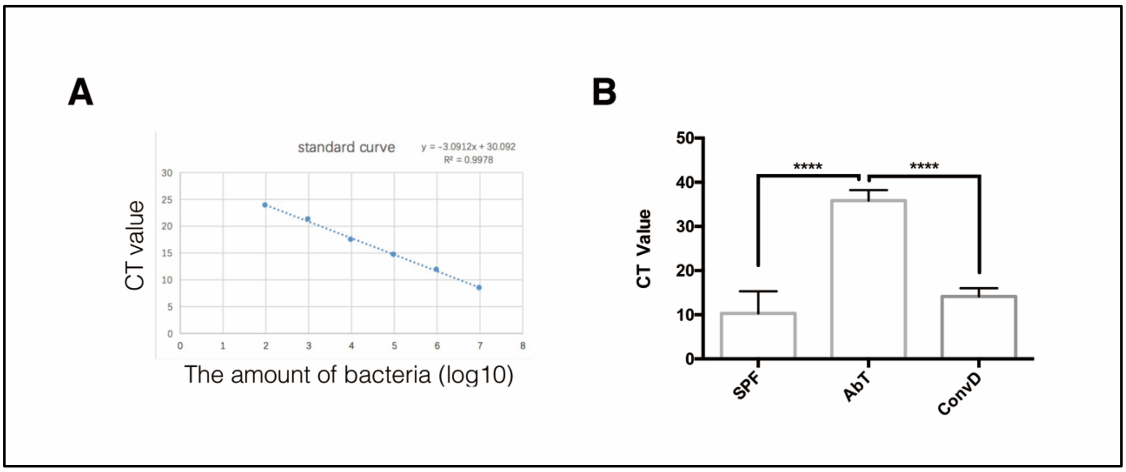


Fig S1. Validation of bacterial depletion efficiency after antibiotic treatment. (A) Standard curve of CT value vs the amount of bacteria. (B)Genomic DNA was isolated from equal amounts of fecal material of SPF, AbT and ConvD mice.16S rRNA gene expression of total bacteria were determined by qPCR, using universal 16S rRNA primers. ****P < 0.0001.

**Figure S2**

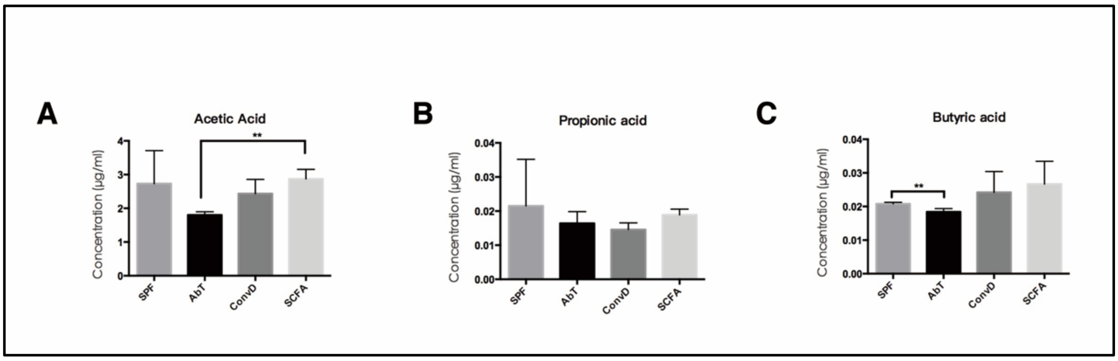


Fig S2. Serum concentration of SCFAs showed decreased levels of acetic, propionic and butyric acid in AbT mice. **P < 0.01.
